# Healthcare Reform and the Next Generation: United States Medical Student Attitudes toward the Patient Protection and Affordable Care Act

**DOI:** 10.1371/journal.pone.0023557

**Published:** 2011-09-13

**Authors:** Kristin M. Huntoon, Colin J. McCluney, Christopher A. Scannell, Elizabeth A. Wiley, Richard Bruno, Allen Andrews, Paul Gorman

**Affiliations:** 1 New York College of Osteopathic Medicine, Old Westbury, New York, United States of America; 2 University of Washington School of Medicine, Seattle, Washington, United States of America; 3 University of Southern California Keck School of Medicine, Los Angeles, California, United States of America; 4 School of Medicine, George Washington University, Washington, D.C., United States of America; 5 School of Medicine, Oregon Health and Science University, Portland, Oregon, United States of America; 6 American Medical Student Association, Reston, Virginia, United States of America; Yale University School of Medicine, United States of America

## Abstract

**Context:**

Over one year after passage of the Patient Protection and Affordable Care Act (PPACA), legislators, healthcare experts, physicians, and the general public continue to debate the implications of the law and its repeal. The PPACA will have a significant impact on future physicians, yet medical student perspectives on the legislation have not been well documented.

**Objective:**

To evaluate medical students' understanding of and attitudes toward healthcare reform and the PPACA including issues of quality, access and cost.

**Design, Setting, and Participants:**

An anonymous electronic survey was sent to medical students at 10 medical schools (total of 6982 students) between October–December 2010, with 1232 students responding and a response rate of 18%.

**Main Outcome Measures:**

Medical students' views and attitudes regarding the PPACA and related topics, measured with Likert scale and open response items.

**Results:**

Of medical students surveyed, 94.8% agreed that the existing United States healthcare system needs to be reformed, 31.4% believed the PPACA will improve healthcare quality, while 20.9% disagreed and almost half (47.7%) were unsure if quality will be improved. Two thirds (67.6%) believed that the PPACA will increase access, 6.5% disagreed and the remaining 25.9% were unsure. With regard to containing healthcare costs, 45.4% of participants indicated that they are unsure if the provisions of the PPACA will do so. Overall, 80.1% of respondents indicated that they support the PPACA, and 78.3% also indicated that they did not feel that reform efforts had gone far enough. A majority of respondents (58.8%) opposed repeal of the PPACA, while 15.0% supported repeal, and 26.1% were undecided.

**Conclusion:**

The overwhelming majority of medical students recognized healthcare reform is needed and expressed support for the PPACA but echoed concerns about whether it will address issues of quality or cost containment.

## Introduction

On March 23, 2010, President Obama signed into the law the Patient Protection and Affordable Care Act (“PPACA”). This piece of legislation has been heralded as the biggest change in our healthcare system since Medicare and Medicaid were enacted in 1965 [1]–[3]. With such historic reform underway, public opinion is divided on the law and its implications.

Substantial polling of the general public has been conducted. According to a Pew Research Center poll, which was completed shortly prior to passage of the PPACA, 48% of Americans opposed the legislation while 38% favored it [4]. Simultaneously, a Kaiser Family Foundation (KFF) poll showed 46% of Americans support the bill and 42% oppose it [5]. National polls have also reflected what Americans view as the strengths and weaknesses of the bill. Some view the PPACA as an opportunity to care for the uninsured or lower-income groups or provide needed oversight of the health insurance industry, while others believe the budget deficit will increase under the new law or create too much government involvement [6]. All of these polls have sought to capture public perceptions of the PPACA and track changing opinions over time.

Physician attitudes regarding health care reform have been previously documented [7], [8]. There has not, however, been a similar drive to evaluate the attitudes of future physicians, who will enter practice during or shortly after the implementation of many of the provisions outlined in the legislation.

In assessing medical student attitudes toward healthcare reform and universal coverage, limited recent studies have been conducted but have demonstrated that medical students believe expansion of healthcare with access to all regardless of ones ability to pay for services should be a priority in any healthcare legislation [9].

Although medical schools have a responsibility to train future physicians by teaching the basic science of pathology and the art of performing history and physical examinations, they are also responsible for reinforcing the importance of medical professionalism and providing exposure to health policy that may cultivate future healthcare leaders and advocates. However, little data has been collected on how medical students perceive the need for health policy understanding or their support for legislation like the PPACA [10]–[15].

In the context of continuing controversy surrounding implementation of the PPACA, the goals of this study were to assess the opinions of medical students on the need for healthcare reform; their level of understanding and support of the major provisions of the PPACA to improve cost containment, access, and quality; and their attitude toward repealing the PPACA.

## Methods

### Survey Design and Testing

Study investigators designed the survey instrument based on existing literature [9] and investigator hypotheses. An online survey program was used to administer the survey (SurveyMonkey; Portland, Oregon). Demographic information was collected on respondents' gender, school, year in medical school, age, race, ethnic, and political self-identification, state in which the respondent was registered to vote, specialty interest, and medical student organization membership. In addition, the survey contained eight five-point Likert scale questions (strongly disagree, disagree, undecided, agree, or strongly agree). These questions were designed to collect medical students' self-assessment of understanding, knowledge, and attitudes toward the PPACA. This study was conducted with approval of the Oregon Health & Science University Institutional Review Board.

### Population

The electronic, anonymous survey was sent to medical students who were currently enrolled at one of ten accredited allopathic or osteopathic United States medical schools. Student coordinators were recruited at the following medical schools: Albany Medical College, George Washington University School of Medicine, Johns Hopkins School of Medicine, New York College of Osteopathic Medicine, Oregon Health and Science University, University of California at San Francisco School of Medicine, University of Iowa Carver College of Medicine, University of Southern California Keck School of Medicine, University of Washington School of Medicine, and Vanderbilt University School of Medicine. Email invitations to complete the survey were sent by student coordinators to all students enrolled at each of these medical schools at the time of the study, using school-maintained listservs. Participation in the study was completely voluntary and no incentives were offered for participation. A fact sheet was included with the email that explained the purpose of the study and provided contact information for the senior author. The email contained an embedded link to the survey instrument hosted at SurveyMonkey.com (Portland, OR; Pro account was purchased), and students used the link to access the survey instrument. Three schools originally slated to participate were excluded as the procedure for survey mailings did not follow procedures as outlined in the IRB proposal (Jefferson Medical College, Rosalind Franklin University, and University of North Carolina School of Medicine).

### Data Collection

All data collected were deidentified so that each survey response could not be linked to any specific individual. To address this limitation, the data were analyzed for discrepancies using Internet Protocol (IP) addresses captured by the online survey program. No identical responses were detected or discarded. To preserve anonymity of respondents, IP addresses were not used for any other purpose.

### Measures and Variables

Response categories were either categorical (gender, medical school, medical school class, race, ethnic, and political self identification, specialty interest, etc.), Likert scale (strongly disagree, disagree, undecided, agree, or strongly agree) or statement choice that best reflected their view (e.g., “I support PPACA but think more reform is needed.”). There were also questions that elicited open-ended text responses (“What type of healthcare system do you favor? If other be specific.”). The measures were calculated percentages of collated responses per category based on the total number of completed responses.

### Statistical Analysis

Simple frequencies were calculated for respondent characteristics and responses to survey questions. The quantitative data were managed and analyzed using Microsoft Excel and JMP (SAS Institute, Cary, NC). A Chi square test was performed to evaluate trends, and statistical significance was defined as a p-value of less than or equal to 0.05. Standard calculations were used in Microsoft Excel to determine 95% confidence intervals [95% CI]. The responses “Strongly agree” and “Agree” were evaluated as “Agree” and the responses “Strongly disagree” and “Disagree” were evaluated as “Disagree.”

## Results

### School and Respondent Characteristics

E-mail invitations were sent to class listservs at the participating schools. After exclusions outlined above, this represented a total enrollment of 6982 based on AAMC data. One thousand two hundred and thirty-two unique responses were received, for a response rate of approximately 18% (1232/6982). Table 1 shows characteristics of the respondents, which are comparable to AAMC data on the national population of students enrolled in accredited medical schools [16]–[19].

**Table 1:** Demographic Characteristics of Respondents.

Characteristics	Respondents N (%)	AAMC data N (%)^a^
**Total**	1232/6982 (17.9%)	79070
**Age** (years)	25.9±3.5	
**Gender** (N (%) female)	634 (51.5%)	37499 (47.4%)
**Year in Training**		
MS1	397 (32.2%)	
MS2	296 (24.0%)	
MS3	281 (22.8%)	
MS4	213 (17.3%)	
Fellow/PhD/MPH	46 (3.7%)	
**Ethnicity and Race self-identification**		
Cuban	4 (0.3%)	585 (0.7%)
Mexican	33 (2.7%)	2058 (2.6%)
Spanish, other	34 (2.8%)	3865 (4.9%)
Not Spanish/Hispanic/Latino	1161 (94.2%)	71446 (90.4%)
American Indian	9 (0.7%)	654 (0.8%)
Asian	192 (15.6%)	17375 (22.0%)
Black, African American	33 (2.7%)	5548 (7.02%)
Native Hawaiian	1 (0.1%)	240 (0.3%)
Other race	67 (5.4%)	3655 (4.6%)
White	925 (75.1%)	47525 (60.1%)
**Political self-identification**		
Very Conservative	12 (1.0%)	
Conservative	159 (12.9%)	
Centrist	209 (17.0%)	
None	124 (10.1%)	
Liberal	555 (45.0%)	
Very Liberal	174 (14.1%)	
**Specialty interest**		
Primary Care (Family Medicine, Internal Medicine, Pediatrics, OB/GYN)	451 (36.6%)	
Internal Medicine Specialty	214 (17.4%)	
General Surgery	45 (3.7%)	
Surgical Sub-Specialty	178 (14.4%)	
Other/Undecided	345 (28.0%)	
**Number of Organizational Memberships**		
0	401 (32.5%)	
1	384 (31.2%)	
2+	447 (36.3%)	

a = Data was only obtained for gender and race comparisons.

### Understanding of the PPACA

As a self-evaluation of knowledge regarding the PPACA, the respondents were asked to assess their own understanding of the bill and its major provisions. Approximately 53.9% agreed [p<0.001, 95% CI 51.1%–56.7%] that they understand the major provisions whereas 30.2% [95% CI 27.7%–32.8%] disagreed. The remaining 15.9% [95% CI 14.0%–18.1%] were undecided (Table 2).

**Table 2:** Responses about the Patient Protection and Affordable Care Act (PPACA).

		Response (%)	95% CI	p value for trend
**I understand the major provisions of recently enacted health care reform legislation (PPACA).**	Strongly Agree/Agree	664 (53.9%)	51.1%–56.7%	p<0.001
	Undecided	196 (15.9%)	14.0%–18.1%	
	Strongly Disagree/Disagree	372 (30.2%)	27.7%–32.8%	
**The American health care system as it exists today needs to be reformed.**	Strongly Agree/Agree	1168 (94.8%)	93.4%–95.9%	p<0.001
	Undecided	45 (3.7%)	2.7%–4.9%	
	Strongly Disagree/Disagree	19 (1.5%)	1.0%–2.4%	
**PPACA will improve health care quality.**	Strongly Agree/Agree	387 (31.4%)	28.9%–34.1%	p<0.001
	Undecided	588 (47.7%)	44.9%–50.5%	
	Strongly Disagree/Disagree	257 (20.9%)	18.7%–23.2%	
**PPACA will expand access to health care.**	Strongly Agree/Agree	833 (67.6%)	64.9%–70.2%	p<0.001
	Undecided	319 (25.9%)	23.5%–28.4%	
	Strongly Disagree/Disagree	80 (6.5%)	5.2%–8.0%	
**PPACA will contain health care costs.**	Strongly Agree/Agree	229 (18.6%)	16.5%–20.9%	p<0.001
	Undecided	559 (45.4%)	42.6%–48.2%	
	Strongly Disagree/Disagree	444 (36.0%)	33.4%–38.8%	
**Which statement best describes your attitude toward recent health care reform legislation?**	I do not support PPACA because it did not go far enough.	75 (6.1%)	4.9%–7.6%	p<0.001
	I do not support PPACA because it went too far.	171 (13.9%)	12.1%–15.9%	
	I support PPACA and think that it went far enough.	27 (2.2%)	1.5%–3.2%	
	I support PPACA but think it went too far.	70 (5.7%)	4.5%–7.1%	
	I support PPACA but think more reform is needed.	889 (72.2%)	69.6%–74.6%	
**Do you support repeal of recent health care reform legislation (PPACA)?**	Yes	185 (15.0%)	13.1%–17.1%	p<0.001
	No	725 (58.8%)	56.1%–61.6%	
	Undecided	322 (26.1%)	23.8%–28.7%	

### The Need for Reform

The overwhelming majority of medical students, 94.8% [95% CI 93.4%–95.9%], agreed that the existing United States healthcare system needs to be reformed; 1.5% [95% CI 1.0%–2.4%] disagreed and 3.7% [95% CI 2.7%–4.9%] of students were undecided about the need for reform (Table 2).

### Health Care Quality and Access

Nearly a third of medical students (31.4% [95% CI 28.9%–34.1%]) believed the PPACA will improve healthcare quality whereas 20.9% [95% CI 18.7%–23.2%] did not agree that it will result in measurable improvements. Nearly half of students, 47.7% [95% CI 44.9%–50.5%], were unsure if healthcare quality will be improved as a result of the PPACA. Two-thirds of students, (67.6% [95% CI 64.9%–70.2]), believed that the PPACA will increase access, whereas only 6.5% [95% CI 5.2%–8.0%] disagreed. One quarter of students (25.9% [95% CI 23.5%–28.4%]) were unsure if the PPACA reforms will result in improved access to care (Table 2).

### Cost-containment

Almost half of participants (45.4% [95% CI 42.6%–48.2%]) indicated that they were unsure if the provisions of the PPACA would contain the rising costs of healthcare. Only 18.6% [95% CI 16.5%–20.9%] of students agreed that the PPACA will contain healthcare costs whereas 36.0% [95% CI 33.4%–38.8%] disagreed (Table 2).

### Support for the PPACA

When medical students were given the opportunity to choose from five preformed statements to most closely describe their opinion of the recently passed legislation, 72.2% [95% CI 69.6%–74.6%] selected “I support PPACA but think more reform is needed.” Including two other supportive choices (2.2% for “I support PPACA and think that it went far enough.” and 5.7% for “I support PPACA but think it went too far.”), the majority of participants (80.1%) [95% CI 77.7%–82.2%] expressed support for the PPACA. Approximately 20.0% [95% CI 17.8%–22.3%] of respondents indicated that they did not support PPACA (6.1% chose “I do not support PPACA because it did not go far enough.” and 13.9% said “I do not support PPACA because it went too far.”). Stating these results differently, of medical students responding, 78.3% [95% CI 75.9%–80.5%] indicated that they felt the PPACA reforms had not gone far enough, while 19.6% [95% CI 17.4%–21.9%] felt that the reforms had gone too far (Table 2).

### To repeal or not to repeal…

When asked about support for repeal of the PPACA, 58.8% [95% CI 56.1%–61.6%] of students were opposed to repeal, 15.0% [95% CI 13.1%–17.1%] supported repeal of the PPACA while 26.1% [95% CI 23.8%–28.7%] were undecided (Table 2).

## Discussion

More than a year after passage of the PPACA, there is still significant uncertainty about its implementation and implications. Numerous studies of the general public and practicing physicians have revealed a lack of consensus with respect to healthcare reform. Polls have shown a wide range of views on whether the PPACA should be repealed. Until now, there have been no comparable surveys of the nation's next generation of physicians. In this first study of the perspectives of medical students, we find some similarities and differences relative to the general public in terms of support for reform in general.

Nearly all of the medical students surveyed (94.8%, [95% CI 93.4%–95.9%]) agree that the current American healthcare system requires reform. Interestingly, this percentage was consistent regardless of how far students had progressed in their medical education, meaning that even prior to significant clinical exposure in the third and fourth years, medical students are well aware of the inadequacies of the current system (Table S1). Additionally, arguments that pro-reform perspectives might be a product of youthful idealism appear to be unfounded as support is steady regardless of age range and may even increase among older students (Table S2). Medical students heavily favor reform of the healthcare system, regardless of political identification or intended specialty (Tables S3, S4). Support for reform is lowest among those who selected “none” for their political self-identification, at 87.9% (109/124) [95% CI 81.0%–92.5%] (Table S3).

Nearly a third of medical students surveyed (30.2% [95% CI 27.7%–32.8%]) felt that they did not understand the major provisions of the PPACA and an additional 15.9% [95% CI 14.0%–18.1%] selected “Undecided” to best describe their understanding (Table 2). These results are similar to those obtained by Deloitte in a poll of the general public at the end of 2010 that found that 38% of Americans admit they are “not at all knowledgeable” about the new legislation [20]. This lack of familiarity with impending changes to the healthcare system is worrisome given that the PPACA, whether in its current form or as amended in the future, will shape the practice of medicine for decades to come. One possible explanation for this phenomenon is inadequate health policy exposure and training at many medical schools. This lack of understanding may also be attributable to inadequate or inaccurate education through the media of both the general public and medical professionals on the details of the PPACA and its implementation.

With respect to improving quality and access, medical student reviews of the PPACA were mixed. Almost half of respondents (47.7% [95% CI 44.9%–50.5%]) were unsure if the reforms would result in improved quality of healthcare, with the remaining respondents roughly split between agreeing (31.4% [95% CI 28.9%–34.1%]) or disagreeing (20.9% [95% CI 18.7%–23.2%]) that it would improve quality. Although assessments of quality can be quite subjective, Gallup has attempted to track how Americans perceive the quality of their healthcare since at least 2002. Perceptions of quality may be affected to a certain extent by factors such as insurance and household income. Those with higher incomes were generally more satisfied with the quality of their care [21]. A March 2010 Kaiser Family Foundation (KFF) poll asked members of the public how they believed the passage of proposed healthcare legislation would affect the quality of their own healthcare: 38% thought quality would remain the same, while 28% felt it would improve quality, and 29% felt quality would worsen. The opinion of medical students is not remarkably different from the respondents to the KFF poll, where the largest proportion of individuals were uncertain, and the remaining responses were fairly evenly split between expecting that quality will be improved and expecting that quality will be worsened [5]. The fact that most medical students are unsure if the PPACA reforms will improve quality may be due to several factors. There may be concerns about the effectiveness of large-scale legislation to affect issues of health quality, or students may get the sense in their clinical exposure that systemic inequities will continue to adversely affect quality despite increased government oversight.

In our survey, 67.6% [95% CI 64.9%–70.2%] of students reported that they believe the PPACA will increase access to care, with only 6.5% [95% CI 5.2%–8.0%] disagreeing (Table 2). In a Deloitte poll, 60% of Americans indicated that they felt access to care would be increased by implementing PPACA [20]. Again, this similarity is likely due to general awareness of well-publicized elements of the reform, such as ending denial of coverage for pre-existing conditions.

Another primary goal for healthcare reform was to “bend the cost curve” and reduce sky-rocketing growth in health expenditures. Over 45.4% [95% CI 42.6%–48.2%] of respondents to our survey indicated that they were unsure if the provisions of PPACA will contain costs. Another 36.0% [95% CI 33.4%–38.8%] felt that the law would not be able to control costs, with only 18.6% [95% CI 16.5%–20.9%] of students holding the opinion that the PPACA will have any positive effect on healthcare spending. In the March 2010 KFF poll, performed before the passage of the final legislation, 31% of respondents felt that their individual cost for healthcare would be reduced by the legislation [5]. However, this does not necessarily reflect generalized cost-savings for the system. The Centers for Medicare and Medicaid Services (CMS) actuaries performed the only available non-partisan analysis of the overall impact of health reform on national health spending. In their projections, health expenditures as a share of the gross domestic product (GDP) would increase from 17.8% in 2010 to 21.0% in 2019. Without the PPACA reforms, health expenditures as a share of GDP were projected to be 20.8% in 2019. This represents an increase of 0.2% of GDP (about $45.8 billion) with reform; however, this small increase in costs (equivalent to less than a tenth of total projected health expenditures for 2019) also includes the 34 million additional Americans who have been estimated to become insured under the PPACA provisions [22]. It is reasonable to see why many students are unsure if the PPACA will control costs, however, given estimates that the legislation will not actually slow the increase in healthcare costs as percentage of GDP even as coverage is increased.

Uncertainty about the effectiveness of elements of the bill aside, 80.1% [95% CI 77.7%–82.2%] of medical student respondents expressed support for the PPACA, selecting a preformed statement that included the phrase “I support PPACA…” (Table 2). Of those who selected supportive statements, 90.2% [95% CI 88.1%–91.9%] felt that reforms had not gone far enough, while only 7.1% [95% CI 5.7%–8.9%] felt that they had gone too far (Table S5). Overall, 78.3% [95% CI 75.9%–80.5%] of respondents, regardless of support for the PPACA, felt that reform efforts were inadequate. This view was the majority opinion regardless of age range, year in school or specialty interest. Perhaps unsurprisingly, the only group where a majority of respondents felt that reform efforts had gone too far was with individuals who self-identified as conservatives −69.0% (118/171) [95% CI 61.7%–75.5%] said too far versus 28.1% (48/171) [95% CI 21.9%–35.2%] who indicated reforms had not gone far enough (Table S3). These general findings, however, are consistent with the high percentage of participants who recognize a need for reform and is consistent with the uncertainty expressed among medical students that the goals of the reform (improved quality, increased access, and cost control) will be adequately achieved by the legislation.

Regardless of the perception of need for further reform, the majority (58.8% [95% CI 56.1%–61.6%]) of students opposed repeal of the PPACA. Another 26.1% [95% CI 23.8%–28.7%] of students were undecided, with only 15.0% [95% CI 13.1%–17.1%] of respondents favoring repeal (Table 2). This is concordant with our other findings that, although medical students have concerns about aspects of the legislation and its ability to address shortcomings of the current system, they recognize a need for reform and are willing to take an imperfect first step in order to improve the system. Other research being done on medical student attitudes has shown the willingness of a majority of medical students to sacrifice their time and earnings in order to care for the underserved population. (Richard Bruno, unpublished data) Interestingly, of the 15.0% who favored repeal, 29.7% (55/185) [95% CI 23.6%–36.7%] indicated that they felt the legislation had not gone far enough with the other 70.3% (130/185) [95% CI 63.3%–76.4%] arguing that it had gone too far (Table S6). What is most remarkable, however, is how medical student opposition to repeal differs from recent polling data for the general public. In the Kaiser Family Foundation's December Health Tracking Poll, one in four respondents (26%) wanted to repeal the law in its entirety; 25% wanted to repeal parts of the law and keep other parts; one in five (21%) wanted to leave the law as it is; and one in five (20%) wanted to expand the law beyond its current footprint [23]. This may be due to a greater level of understanding of the legislation and its implication by medical students or may be due to a different perspective on their role in the delivery and financing of healthcare.

At eighteen percent, our response rate was suboptimal. There are several potential factors that may contribute to the low calculated value for this surveys response rate. First, participant recruitment was via school-maintained listservs, which may not account for all students reported in AAMC data. Second, there was no mechanism to confirm that e-mail notifications were received or read by recipients. Third, participation in the study was voluntary and no incentives were offered for participation. Despite the lower response rate, our sample size was fairly large, providing significant power for analysis, and our respondent characteristics appear to closely mirror the medical student demographics reported by the AAMC. It is possible that, as part of a voluntary survey, the respondent population may be biased toward individuals that are more likely to be involved in organizations working on healthcare and policy issues or personally interested in these issues. Upon further analysis, however, there is no statistical difference in responses of individuals who reported no organizational memberships compared to responses of those who reported two or more organizational memberships (Table S7). When analyzing a volunteer survey, attempts should be made to limit confounding factors such that the data is representative of the population as a whole. We attempted to limit any bias including age, year, race, political identification and other possible sources. While there is no mechanism for us to assess our success in limiting bias, based on our respondent demographics, our sample size, and the option for free-text comments regarding PPACA (not included in this report), we believe this sample is a representative group. Nevertheless, we recognize that there are limitations to voluntary surveys and as such it is possible that our data may contain respondent bias towards students with a greater level of interest in health policy or PPACA.

There are some challenges with comparing our survey results to polls with different question wordings; we have attempted to find polls that match the spirit of our survey questions and have included exact wording where applicable. While our data show provide signicant insight into the views of medical students toward health reform and the PPACA, the surveys did not provide respondents an opportunity to express in more detail what additional elements of reform they would have liked to see or which specific aspects of the PPACA they supported or did not support. This would be an interesting and valuable area for further study, as medical students and other health professional students represent the future of healthcare in the United States. Based on the lack of clear understanding of the major provisions of PPACA reported by medical students in our study, it would be beneficial to further investigate how much education medical students received about the PPACA from their medical schools (or from other organizations). Improved understanding of any deficits in this part of training could help lead to improvements in how health policy topics are incorporated into medical school curricula.

Overall, these results give insight into the medical student perspective on healthcare reform and specifically on the PPACA. In summary, the majority of medical students are globally supportive of the PPACA while recognizing that it is not clear if its lofty goals will be realized. Medical students continue to favor strong reform of the United States healthcare system, with a majority believing that current reform efforts have been inadequate and that further steps are needed to salvage our system.

## Supporting Information

Table S1 1Participants were given a choice of five pre-formed statements: “I do not support PPACA because it did not go far enough”, “I do not support PPACA because it went too far”, “I support PPACA and think that it went far enough”, “I support PPACA but think it went too far”, or “I support PPACA but think more reform is needed.” These results were parsed into “I support…” or “I do not support…” for one analysis and “…it did not go far enough”, “…it went far enough” or “…it went too far” for the other analysis.
(DOCX)

Table S2 1Respondents ranging in age from 21–24 years. 2Respondents ranging in age from 30–53 years. 3Participants were given a choice of five pre-formed statements: “I do not support PPACA because it did not go far enough”, “I do not support PPACA because it went too far”, “I support PPACA and think that it went far enough”, “I support PPACA but think it went too far”, or “I support PPACA but think more reform is needed.” These results were parsed into “I support…” or “I do not support…” for one analysis and “…it did not go far enough”, “…it went far enough” or “…it went too far” for the other analysis.
(DOCX)

Table S3 1Participants were given a choice of five pre-formed statements: “I do not support PPACA because it did not go far enough”, “I do not support PPACA because it went too far”, “I support PPACA and think that it went far enough”, “I support PPACA but think it went too far”, or “I support PPACA but think more reform is needed.” These results were parsed into “I support…” or “I do not support…” for one analysis and “…it did not go far enough”, “…it went far enough” or “…it went too far” for the other analysis.
(DOCX)

Table S4 1Participants were given a choice of five pre-formed statements: “I do not support PPACA because it did not go far enough”, “I do not support PPACA because it went too far”, “I support PPACA and think that it went far enough”, “I support PPACA but think it went too far”, or “I support PPACA but think more reform is needed.” These results were parsed into “I support…” or “I do not support…” for one analysis and “…it did not go far enough”, “…it went far enough” or “…it went too far” for the other analysis.
(DOCX)

Table S5 1Participants were given a choice of five pre-formed statements: “I do not support PPACA because it did not go far enough”, “I do not support PPACA because it went too far”, “I support PPACA and think that it went far enough”, “I support PPACA but think it went too far”, or “I support PPACA but think more reform is needed.” These results were parsed into “I support…” or “I do not support…” for the purposes of this analysis. 2Participants were given a choice of five pre-formed statements: “I do not support PPACA because it did not go far enough”, “I do not support PPACA because it went too far”, “I support PPACA and think that it went far enough”, “I support PPACA but think it went too far”, or “I support PPACA but think more reform is needed.” These results were parsed into “…it did not go far enough”, “…it went far enough” or “…it went too far” for the purposes of this analysis.
(DOCX)

Table S6 1Participants were given a choice of five pre-formed statements: “I do not support PPACA because it did not go far enough”, “I do not support PPACA because it went too far”, “I support PPACA and think that it went far enough”, “I support PPACA but think it went too far”, or “I support PPACA but think more reform is needed.” These results were parsed into “I support…” or “I do not support…” for one analysis and “…it did not go far enough”, “…it went far enough” or “…it went too far” for the other analysis.
(DOCX)

Table S7 1Participants were given a choice of five pre-formed statements: “I do not support PPACA because it did not go far enough”, “I do not support PPACA because it went too far”, “I support PPACA and think that it went far enough”, “I support PPACA but think it went too far”, or “I support PPACA but think more reform is needed.” These results were parsed into “I support…” or “I do not support…” for one analysis and “…it did not go far enough”, “…it went far enough” or “…it went too far” for the other analysis.
(DOCX)
