# Supplementary material for: Healthcare Reform and the Next Generation: United States Medical Student Attitudes toward the Patient Protection and Affordable Care Act
Source: PLoS One. 2011 Sep 13;6(9):e23557. doi: 10.1371/journal.pone.0023557 (PMC3172206; doi:10.1371/journal.pone.0023557)
Supplement: Table S5 — 1Participants were given a choice of five pre-formed statements: “I do not support PPACA because it did not go far enough”, “I do not support PPACA because it went too far”, “I support PPACA and think that it went far enough”, “I support PPACA but think it went too far”, or “I support PPACA but think more reform is needed.” These results were parsed into “I support…” or “I do not support…” for the purposes of this analysis. 2Participants were given a choice of five pre-formed statements: “I do not support PPACA because it did not go far enough”, “I do not support PPACA because it went too far”, “I support PPACA and think that it went far enough”, “I support PPACA but think it went too far”, or “I support PPACA but think more reform is needed.” These results were parsed into “…it did not go far enough”, “…it went far enough” or “…it went too far” for the purposes of this analysis. (DOCX) [file pone.0023557.s005.docx]

**Table S5: Responses by Support of PPACA^1^**

|  | | **I support PPACA… (n=986)** | | **I do not support PPACA… (n=246)** | |
| --- | --- | --- | --- | --- | --- |
|  |  | **Responses (%)** | **95% CI** | **Responses (%)** | **95% CI** |
| **I understand the major provisions of recently enacted health care reform legislation (PPACA).** | Strongly Agree / Agree | 535 (54.3%) | 51.1% - 57.3% | 129 (52.4%) | 46.2% - 58.6% |
|  | Undecided | 159 (16.1%) | 14.0% - 18.6% | 37 (15.0%) | 11.1% - 20.0% |
|  | Strongly Disagree / Disagree | 292 (29.6%) | 26.8% - 32.5% | 80 (32.5%) | 27.0% - 38.6% |
| **The American health care system as it exists today needs to be reformed.** | Strongly Agree / Agree | 535 (54.3%) | 51.1% - 57.3% | 129 (52.4%) | 46.2% - 58.6% |
|  | Undecided | 159 (16.1%) | 14.0% - 18.6% | 37 (15.0%) | 11.1% - 20.0% |
|  | Strongly Disagree / Disagree | 292 (29.6%) | 26.8% - 32.5% | 80 (32.5%) | 27.0% - 38.6% |
| **PPACA will improve health care quality.** | Strongly Agree / Agree | 378 (38.3%) | 35.4% - 41.4% | 9 (3.7%) | 1.9% - 6.8% |
|  | Undecided | 506 (51.3%) | 48.2% - 54.4% | 82 (33.3%) | 27.7% - 39.4% |
|  | Strongly Disagree / Disagree | 102 (10.3%) | 8.6% - 12.4% | 155 (63.0%) | 56.8% - 68.8% |
| **PPACA will expand access to health care.** | Strongly Agree / Agree | 738 (74.8%) | 72.0% - 77.5% | 95 (38.6%) | 32.8% - 44.8% |
|  | Undecided | 229 (23.2%) | 20.7% - 26.0% | 90 (36.6%) | 30.8% - 42.8% |
|  | Strongly Disagree / Disagree | 19 (1.9%) | 1.2% - 3.0% | 61 (24.8%) | 19.8% - 30.6% |
| **PPACA will contain health care costs.** | Strongly Agree / Agree | 215 (21.8%) | 19.3% - 24.5% | 14 (5.7%) | 3.4% - 9.3% |
|  | Undecided | 512 (51.9%) | 48.8% - 55.0% | 47 (19.1%) | 14.7% - 24.5% |
|  | Strongly Disagree / Disagree | 259 (26.3%) | 23.6% - 29.1% | 185 (75.2%) | 69.4% - 80.2% |
| **Which statement best describes your attitude toward recent health care reform legislation?^2^** | …it did not go far enough / …more reform is needed | 889 (90.2%) | 88.1% - 91.9% | 75 (30.5%) | 25.1% - 36.5% |
|  | ….it went far enough | 27 (2.7%) | 1.9% - 4.0% | 0 (0.0%) |  |
|  | …it went too far | 70 (7.1%) | 5.7% - 8.9% | 171 (69.5%) | 63.5% - 74.9% |
| **Do you support repeal of recent health care reform legislation (PPACA)?** | Yes | 52 (5.3%) | 4.0% - 6.9% | 133 (54.1%) | 47.8% - 60.2% |
|  | No | 677 (68.7%) | 65.7% - 71.5% | 48 (19.5%) | 15.0% - 24.9% |
|  | Undecided | 257 (26.1%) | 23.4% - 28.9% | 65 (26.4%) | 21.3% - 32.3% |
